# Residential exposure to chlorinated hydrocarbons from groundwater contamination and the impairment of renal function-An ecological study

**DOI:** 10.1038/srep40283

**Published:** 2017-01-09

**Authors:** Hui-Ming Chen, Ming-Tsang Wu

**Affiliations:** 1 Department of Public Health, College of Health Sciences, Kaohsiung Medical University, Kaohsiung, Taiwan; 2 Department of Family Medicine and Occupational Medicine, Kaohsiung Chang-Gang Memorial Hospital, Kaohsiung, Taiwan; 3 Research Center of Environmental Medicine, Kaohsiung Medical University, Kaohsiung, Taiwan; 4 Department of Family Medicine, Kaohsiung Medical University Hospital, Kaohsiung Medical University, Kaohsiung, Taiwan; 5 Center of Environmental and Occupational Medicine, Kaohsiung Municipal Hsiao-Kang Hospital, Kaohsiung Medical University, No.482, Shanming Road, Kaohsiung 812, Taiwan

## Abstract

Groundwater pollution from the petrochemical industry causes serious deterioration of soil and groundwater quality and impacts on human health worldwide. However, few studies have examined the effect of residential exposure to petrochemical chlorinated hydrocarbon-contaminated groundwater on renal function impairment in humans. We conducted an ecological study to investigate the two. A polyvinyl chloride (PVC) plant was located in one of the six villages, the study area, in Kaohsiung city of southwestern Taiwan. Based on the direction of groundwater flow and previous groundwater measurements of chlorinated hydrocarbons from Taiwan Environmental Protection Bureau, we divided the six villages into highly-polluted villages, moderately-polluted villages, and a non-polluted village. All inhabitants in those six villages were invited to receive free health examinations between May-June, 2010. In total, 4,432 study subjects ≥18 yrs old were analyzed. Compared to those in the non-polluted village, subjects in highly-polluted villages had 1.89- and 1.46-fold the risk of impaired estimated glomerular filtration rate (eGFR) and proteinuria (95% CI = 1.15–1.85 and 1.09–3.28, respectively) after adjusting for other covariates. Given this relative large sample size, we found that groundwater chlorinated hydrocarbon pollution can cause kidney damage in adults.

Groundwater pollution from the petrochemical industry has emerged as an important public and health concern in many countries. Groundwater pollution can potentially cause serious deterioration of soil and groundwater quality^1^,^2^. Because groundwater pollution from petrochemical chlorinated hydrocarbons may travel through soils easily, it can seep into underground drinking water supplies and stay there for months. Residents in such areas where groundwater is the main source of drinking water have been reported to be at increased risk of both acute symptoms such as diarrhea, eye irritation, and sleepiness as well as chronic illnesses such as respiratory diseases, seizures, and a variety of cancers in the respiratory, gastrointestinal, and genito-urinary organs^3^,^4^,^5^,^6^,^7^,^8^,^9^,^10^. In addition, some chlorinated hydrocarbons such as vinyl chloride (VC), trichloroethylene (TCE), and perchloroethylene (PCE) may cause human renal toxicity. The possible mechanisms underlying the damage of kidney caused by chlorinated hydrocarbons are probably due to the disruption of the immune complex system of the kidney and injury to proximal renal tubules which increases the excretion of high molecular weight proteins in urine^11^,^12^.

Taiwan’s population density is high, and thus many of its residents live in close proximity to petrochemical industrial complexes^13^. Although occupational epidemiological studies have reported that several chlorinated hydrocarbons can cause renal damage in the workplaces^14^,^15^,^16^, little is known about environmental or residential exposure to contaminated groundwater and impaired human kidney function. Thus, we performed an ecological study of this possible link in Kaohsiung City, a heavily industrialized city in Taiwan. The study area consisted of six villages, where one large polyvinyl chloride (PVC) factory is situated in one village of them, in northern Kaohsiung City (Fig. 1). We provided free health examinations for residents of those six villages in May-June, 2010, at which time we collected data related to kidney impairment.

## Results

### Data of volatile organic compounds (VOCs) from groundwater

**Figure 1:**
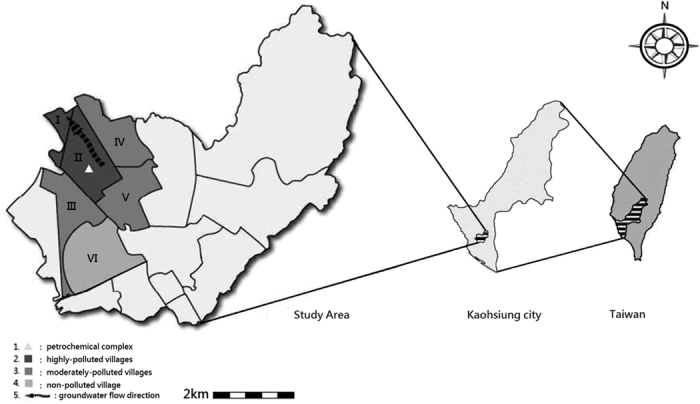
The study area and the direction of groundwater flow. The source map was generated from OpenStreetMap online platform (http://openstreetmap.tw/osmtw) (©OpenStreetMap) and modified by Microsoft Paint version 5.1 with illustrator CC(2014). The cartography in the OpenStreetMap map tiles is licensed under CC BY-SA (http://www.openstreetmap.org/copyright). The licence terms can be found on the following link: http://creativecommons.org/licenses/by-sa/2.0/. Microsoft Paint version 5.1 → base map creation URL: http://en.softonic.com/s/microsoft-paint-version-5.1/windows-xp illustrator CC(2014) → base map modification URL:http://getintopc.com/softwares/graphic-design/adobe-illustrator-cc-2014-free-download/.

Groundwater flowed from southeast to northwest (Fig. 1). The concentrations of vinyl chloride (VC), trichloroethylene (TCE), and perchloroethylene (PCE) in groundwater from some of the wells in highly polluted villages were detectable at levels beyond pollution control standards established by Kaohsiung City Environmental Protection Bureau (Table 1). The lower concentrations of VC, TCE, and PCE were also detected in the moderately polluted village. Besides, some of wells were found to have other volatile organic compounds (VOCs), including chloroform (CHCL3), cis-1,2-dichloroethene (cis-1,2-DCE), 1,1-dichloroethylene (1,1-DCE), 1,1-dichloroethane (1,1-DCEA), 1,2-dichloroethane (1,2-DCEA), dichloromethane (DCM), and toluene (TLN). None the VOCs we study could be detected in groundwater from the one well in village VI, the non-polluted village.

### Study participants

**Figure 2:**
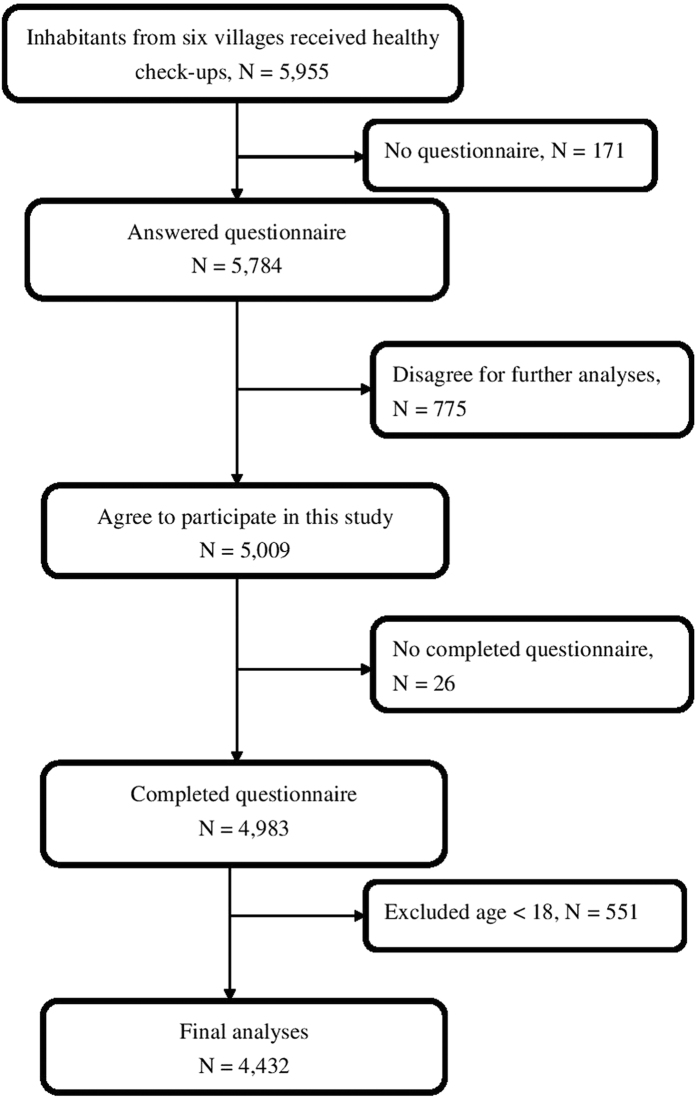
Study flowchart.

Totally 5,955 village inhabitants received health examinations. Of those, 5,009 agreed to participate in this study. After excluding subjects with incomplete questionnaires (N = 26) and those below 18 yrs old (N = 551), we were left with the data of 4,432 subjects to analyze (Fig. 2).

### Demographics and clinical variables among the three exposure groups

Subjects living in the highly polluted villages were older and less educated, and among them, there were more smokers, betel nut chewers, and more people with a history of diabetes, compared to the other villages (Table 2). The highest mean systolic and diastolic blood pressures were found in subjects in the highly polluted villages, followed by the moderately polluted village, and lowest in the non-polluted village (eTable 1).

### Renal functional impairment in three exposure groups

Renal abnormality rates were highest in highly polluted villages and lowest in the non-polluted village (Table 3). Compared to those in the non-polluted village, subjects in highly polluted village had 1.48- and 2.72-fold the risk of proteinuria and estimated glomerular filtration rate (eGFR) impairment, respectively (Table 4). After adjusting for all covariates, the results remained similar (adjusted odds ratio (OR) = 1.46, 95% confidence interval (CI) = 1.15–1.85 for proteinuria and adjusted OR = 1.89, 95% CI = 1.09–3.28 for eGFR impairment). Among the subjects in the moderately-polluted villages, eGFR impairment was significant (adjusted OR = 1.74, 95% CI = 1.02–2.97), but proteinuria was not (adjusted OR = 0.92, 95% CI = 0.73–1.15), compared to those in the non-polluted village (Table 4). Proteinuria was most significant for village I (adjusted OR = 1.70, 95% CI = 1.25–2.32) and eGFR impairment most significant in villages II and III+ IV (adjusted OR = 1.95 and 2.38, 95% CI = 1.01–3.74 and 1.04–5.43) (eTable 3).

In the subgroup analyses, proteinuria was significant in subjects under 65 yrs old and in those with no history diabetes (eTable 4 and eTable 5). We did not find any significant differences in eGFR impairment by age and history of diabetes.

We found no significant correlation between serum renal function indicators (blood urea nitrogen, creatinine, and uric acid) and exposure (eTable 6). These results remained insignificant in the subgroup analyses (data not shown).

## Discussion

The study found that subjects residing in communities with chlorinated hydrocarbon- contaminated groundwater to be at significantly higher risk of proteinuria and impaired eGFR than those residing in communities without contaminated groundwater. The risk of proteinuria was particularly significant in subjects who were under 65 years old and in those with no history of diabetes. However, we found no significant group differences in serum indicators of renal function, suggesting those indicators may not be as sensitive as urine indicators.

Many environmental epidemiological studies, including some from Taiwan, have linked volatile chlorinated hydrocarbon-polluted groundwater with risk and death due to a variety of gastrointestinal, liver, and urinary system cancers^4^,^5^,^6^,^7^,^8^. In addition, some studies have also reported an association between this pollution and eye irritation, respiratory diseases, diarrhea, sleepiness, and seizures^9^,^10^.

Some occupational studies have linked exposure to chlorinated hydrocarbons such as VC, TCE, and PCE in the workplace to nephropathy in workers^11^,^12^,^17^,^18^,^19^,^20^. One cohort study performed in a cardboard factory in Germany found an increased incidence of renal cell cancer in workers exposed to TCE^21^. The standardized incidence ratio (SIR) for renal cancer were found to be higher in workers exposed to TCE compared to the general population (8.0; 95% CI = 2.6–18.6). In a case–control study performed by Vamvakas and colleagues in the same area, the odds ratio (OR) of renal cancer in those exposed to TCE was significantly increased (10.8; 95% CI = 3.4–34.8), compared to those not exposed to TCE, after adjusting for age, chronic intake of diuretics, smoking, BMI and blood pressure^22^. A recent meta-analysis of twenty-three studies concluded that there was a probable relationship between TCE exposure and kidney cancer^14^. Across all studies meeting that study’s inclusion criteria (n = 23), the summary relative risk (RR) for kidney cancer was 1.42 (95% CI = 1.17–1.77), with heterogeneity present (test for heterogeneity: *P* = 0.001). Verplanke *et al*. conducted a study to investigate the effects of PCE exposure on the kidney status in 82 exposed and 19 nonexposed workers from four dry-cleaning shops in the Netherlands. In that study, effects on renal tubular function were examined by measuring urinary *N*-acetyl-β-D- glucosaminidase, β-galactosidase, alanine aminopeptidase, and retinol-binding protein. Glomerular function was monitored by measuring total protein and albumin in the urine. Retinol-binding protein was the only parameter found to be increased in the group exposed to PCE, compared to not exposed (75.4 *vs.* 41.6 mg/g of creatinine)^15^. These results suggest that occupational exposure to PCE may have a minor effect on tubular function. A recent 8-year follow-up cohort study of mortality in 1,704 dry cleaning workers in four cities in the United States found that overall cancer deaths were significantly greater in this cohort (standardized mortality ratio (SMR) = 1.22; 95% CI = 1.09–1.36). That study also found hypertensive end stage renal disease (ESRD) morbidity to be significantly elevated in their entire cohort (SIR = 1.98; 95% CI = 1.11 to 3.27) and among workers employed only in PCE-using dry cleaning shops for ≥5 years^16^.

The possible mechanisms underlying the damaged kidney caused by chlorinated hydrocarbons include the disruption of the immune complex system of the kidney resulting in immunocomplex nephritis^11^ and injury to proximal renal tubules which increases the excretion of high molecular weight proteins in urine causing nephrotoxicity^12^. The metabolites of TCE or PCE derived from the glutathione (GSH) conjugation pathway are responsible for most the effects on the kidney. The key metabolites of TCE possibly associated with nephrotoxicity and tumorigenesis include S-(1,2-dichlorovinyl) glutathione (DCVG), S-(1,2-dichlorovinyl)-L-cysteine (DCVC) and DCVC sulfoxide^23^. Similarly, the metabolites of PCE possibly associated with renal toxicity and tumorigenesis include S-(1,2,2- trichlorovinyl) glutathione (TCVG) and S-(1,2,2-trichlorovinyl)-L-cysteine (TCVC)^24^. The four major mechanisms through which TCE or PCE may cause renal toxicity are peroxisome proliferation, α2u-globulin nephropathy, genotoxicity, and acute or chronic nephrotoxicity. The latter involves the induction oxidative stress, mitochondrial dysfunction, protein alkylation, and deoxyribonucleic acid (DNA) alkylation. Several modes of action may contribute importantly to nephrotoxicity induced by chlorinated hydrocarbons, and different modes of action or combinations of modes may factor into the nephrotoxicity caused by chlorinated hydrocarbons at high or low doses. If cells are exposed to very high doses of chlorinated hydrocarbons that cause extreme mitochondrial dysfunction, it is likely that the tissue will not be able to undergo repair and proliferation. At lower doses, in contrast, it is likely that mild changes in mitochondrial function and oxidative stress as well as selective alkylation of protein and DNA will occur, and that these effects will lead to changes in homeostatic processes in the cell that will ultimately alter gene expression and cell growth.

Humans can be exposed to polluted groundwater through ingestion, inhalation, or absorption through the skin. Although ingestion of drinking water is thought to be a primary route of exposure^25^, this study found that only seven percent of the residents in these communities used groundwater as a source of drinking water. In addition, Taiwanese people usually boil tap water before drinking it. One previous study has reported that chlorinated hydrocarbons in water decrease significantly with increases in temperature and their concentrations are negligible after one minute of boiling^26^. Inhalation from indoor air is another route^27^. People can be exposed to ambient chlorinated hydrocarbons from ground water when showering, washing vegetables and dishes or performing other cleaning activities daily. It has been estimated that more than half of the total inhalation exposure comes from showering in Taiwan^26^. Dermal absorption is another route and this can occur when showering and washing. Brown and colleagues suggest that dermal uptake of compounds occur mainly through passive diffusion and estimate that sixty-four percent (range 29–91%) of the average uptake of water-borne VOCs come from skin absorption in humans^28^. Therefore, the main routes of exposure to chlorinated hydrocarbons in groundwater are dermal absorption and inhalation, though, according to one study, overall hazard for inhalation is ten times higher than dermal absorption^26^.

This study has several limitations. One limitation is that, although we encouraged all residents in the study areas to receive health examinations for free, some of them may have been more willing to participate than others, especially villagers who were sick or living in the highly polluted villages. This bias might result in some overestimations in our results. However, because they did not know the hypothesis of the study, volunteer bias was probably reduced. Another limitation is that we did not collect individual exposure data, which might lead to over and underestimations. Still another limitation is that we did not collect data regarding individual work history and ambient exposure data. Thus, it was impossible to distinguish between occupational and environmental exposure to chlorinated hydrocarbons. Finally, because this study is cross-sectional, causality between chlorinated hydrocarbons exposure from groundwater and renal function impairment cannot be directly inferred.

This study found a significant association between groundwater polluted with chlorinated hydrocarbons and impaired kidney function. Further research is needed to establish the personal exposure dose and to elucidate the possible dose-response relationship.

## Materials and Methods

### Study area

Kaohsiung City, a harbor city located on the southwestern coast of Taiwan, is home to a number of large oil and petrochemical industrial zones^29^. As can be seen in Fig. 1, one large PVC plant is located in this city in one community, which called village II. This village is surrounded by other four villages (village I, III, IV, and V) (Fig. 1). The PVC plant, built in 1972, produces 28,800 tons of PVC annually. Later, expanding its chemical production, this plant established a vinyl chloride monomer (VCM) processing area in 1975 and a hydrochlorofluorocarbon (HCFC) processing area in 1990. According to Taiwan’s Environmental Protection Administration, this PVC plant is Taiwan’s greatest producer of organic chlorinated hydrocarbon waste, including: 1,1-dichloroethene (1,1-DCEA), 1,2-dichloroethane (1,2-DCEA), vinyl chloride (VC), dichloromethane (DCM), and chloromethane.

Because officials at Kaohsiung City’s Environmental Protection Bureau were concerned that this company might illegally release some of these chemicals underground potentially polluting the underground water in the surrounding villages, they assessed PCE, TCE, and VC, and seven other volatile organic compounds in samples obtained the underground water of these six villages in 2010 (Table 1).

### Subjects and data collection

Kaohsiung City Environmental Protection Bureau assigned one medical center, Kaohsiung Chang-Gung Memorial Hospital (KCGMH), to provide free health examinations for volunteer residents in and around the villages surrounding that petrochemical company. KCGMH, the medical center closest to the study site, offered these free health examinations at the local activities centers of these villages from May 8 to June 21, 2010.

After each volunteer signed a written consent form, he or she was given self-administered questionnaire followed by a health examination. Anyone with household registrations indicating that they had moved out of these villages within the prior two years was also invited to participate in this free health examination. At the time of the health examination, a history of chronic diseases and personal lifetime habits was surveyed, physical examinations were performed, and blood and urine samples were obtained for clinical biochemistry analyses. This study was approved by the institutional review board of KCGMH. The methods were carried out in accordance with The Declaration of Helsinki. Written information was provided and informed consent was obtained from all subjects.

### Questionnaire and physical examination

All the participants were administered a structured questionnaire to collect the detailed information including age, gender, residence, and other sociodemographic characteristics including their use of substances (cigarette, alcohol, and betel quid) and sources of drinking water. Participants were defined as alcohol drinkers, cigarette smokers or betel quid chewers if they regularly consumed any alcoholic beverage ≥1 times per week, smoked ≥10 cigarettes per week, or chewed ≥1 betel quid per day for at least six months^30^.

During the physical examination, body height, body weight, and waist circumference were measured by professional examiners while participants stood in light street clothes. In addition, blood pressure was measured using a standardized mercury column sphygmomanometer and an appropriately sized cuff after the subject had rested for at least 5 min. Two measurements were taken and the average of the two measurements was recorded.

### Blood and urine samples

One-time blood samples were collected in the morning by veni-puncture after fasting at least 8 hr. The blood samples were divided into three: one blood sample for routine blood workup, another blood sample for serum biochemistry such as liver function (glutamate oxaloacetate transaminase (GOT), glutamic-pyruvic transaminase (GPT), r-glutamyl transpeptidase (RGT)), cardiometabolic function (glucose, total cholesterol, triglyceride), thyroid profile (triiodothyronine (T3), thyroxin (T4), thyroid - stimulating hormone (TSH)), and renal function (blood urea nitrogen (BUN), creatinine, and uric acid), hepatitis B antigen (HBsAg), anti-hepatitis C antibody (anti-HCV), and the third blood sample was used to detect tumor markers such as alpha-fetoprotein (AFP), carcinoembryonic antigen (CEA), prostatic specific antigen (PSA), cancer antigen 125 (CA125), and cancer antigen 19–9 (CA19–9). Serum was stored at −80 °C until analysis. All measurements were performed in the central clinical laboratory of at KCGMH.

After the collection of blood sample, a one-spot urine sample was collected to perform a routine urinary analysis and to screen for proteinuria. All urine samples were stored at −20 °C until analysis.

### Definition of kidney function impairment

According to the National Kidney Foundation Kidney Disease Outcomes Quality Initiative (KDOQI) clinical practice guidelines for chronic kidney disease, the definition and staging of chronic kidney disease depends on the assessment of Glomerular Filtration Rate (GFR), proteinuria, and other markers of kidney disease. An equation in Modification of Diet in Renal Disease (MDRD) was used to estimate glomerular filtration rate^31^ (MDRDGFR = 186.3 × Plasma creatinine (mg/dl)^−1.154^ × age (yr)^−0.203^ × (1.212 if black) × 0.742 if female). Participants whose eGFRs were less than 60 ml/min/1.73 m^2^ or who were found to have proteinuria were defined as having impaired kidney function. KCGMH’s clinical cut-off points for BUN, creatinine, and uric acid in serum were used as indicators of renal function. The abnormal values in serum for BUN were >20 mg/dl, creatinine >1.27 mg/dl in males and >1.03 mg/dl in females, and uric acid >8.3 mg/dl.

### Statistical analysis

Based on the site of the PVC plant, the direction of groundwater flow (Fig. 1) and Kaohsiung Environmental Protection Bureau Groundwater measurements (Table 1), the villages were categorized into three exposure groups: highly polluted villages (village I and II), moderately polluted villages (village III, IV, and V), and a non-polluted village (village VI). The demographic characteristics of the subjects and their clinical characteristics between groups were analyzed by either analysis of variance (ANOVA) or Chi-square test to analyze differences in categorical and continuous variables. To examine the association between exposure groups and kidney function impairment (proteinuria or eGFR <60 ml/min/1.73 m^2^), the non-polluted village was compared with the highly and moderately polluted villages before and after adjusting for other covariates that were found to be significant in simple logistic regression. These included age, gender, educational level, marriage, vegetarianism, cigarette smoking, betel quid chewing, alcohol use, and source of drinking water for proteinuria. With the exception of age and sex, the covariates were similar for eGFR. The analyses were rerun and further adjusted for body mass index, diabetes, hypertension, uric acid, and anti-HCV. The same strategies were also applied to serum BUN, creatinine, and uric acid, based on KCGMH clinical cut-off points.

To examine the robustness of the results, data were also sub-grouped by age (<65 or ≥65 yrs) or the history of diabetes (present or absent). All statistical operations were performed using the Statistical Package for the Social Sciences (version 17.0; SPSS Inc, Chicago, IL, USA). A *p*-value of <0.05 was considered significant.

## Additional Information

**How to cite this article**: Chen, H.-M. and Wu, M.-T. Residential exposure to chlorinated hydrocarbons from groundwater contamination and the impairment of renal function-An ecological study. *Sci. Rep.* **7**, 40283; doi: 10.1038/srep40283 (2017).

**Publisher's note:** Springer Nature remains neutral with regard to jurisdictional claims in published maps and institutional affiliations.

## Supplementary Material

Supplementary Information

## Figures and Tables

**Table 1:** The concentrations of certain volatile organic compounds in groundwater of six study villages as measured by Kaohsiung Environmental Protection Bureau.

Village	Well No.	VC	TCE	PCE	Others						
					TLN	1,1-DCE	1,1-DCEA	1,2-DCEA	cis-1,2-DCE	DCM	CHCL3
I	C-03	—1	—	—	1.01	—	—	—	—	—	—
	C-11	—	—	—	—	—	1.5	—	—	—	—
	C-05	35.2^2^	—	—	—	2.78	0.83	3.59	—	—	—
II	A-15	—	—	—	—	—	—	—	1.51	—	—
	I-01	—	11.4^3^	—	-	—	—	—	—	—	—
	Q-06	1.03	—	—	—	—	—	—	—	—	—
III	D-03	4.12^3^	—	—	2.24	—	—	—	—	—	—
	D-10	0.86	—	—	—	—	1.01	—	—	—	—
	E-01	—	—	—	—	—	—	—	—	1.15	—
	E-14	—	—	—	6.59	—	—	—	—	—	—
	E-18	—	—	5.33^3^	—	—	—	—	1.27	—	—
	E-20	—	4.72	28.3^3^	—	—	—	—	1.93	—	—
	L-01	—	—	24.8^3^	—	—	—	—	—	—	—
	L-02	—	—	—	—	—	—	—	—	—	1.07
IV	P-01	—	—	—	—	—	—	—	—	—	—
	L-04	—	—	—	1.14	—	—	—	—	—	—
V	F-01	—	—	—	—	—	—	0.94	—	—	—
	N-01	—	—	—	—	—	1.09	—	—	—	—
VI	K-01	—	—	—	—	—	—	—	—	—	—

Abbreviations: CHCL3 = chloroform; cis-1,2-DCE = cis-1,2-dichloroethene; 1,1-DCE = 1,1-dichloroethylene; 1,1-DCEA = 1,1-dichloroethane; 1,2-DCEA = 1,2-dichloroethane; DCM = dichloromethane; PCE = perchloroethylene; TCE = trichloroethylene, TLN = toluene; VC = vinyl chloride.

^1^“—”Indicates the concentration below the detection limit (μg/L) which was 0.83 for CHCL3, 0.66 for cis-1,2-DCE, 0.62 for 1,1-DCE, 0.67 for 1,1-DCEA, 0.65 for 1,2-DCEA, 0.85 for DCM, 0.75 for PCE, 0.61 for TCE, 0.68 for TLN, and 0.69 for VC.

^2^The concentration larger than second category of groundwater pollution control standards (μg/L) which were 1,000 for CHCL3, 700 for cis-1,2-DCE, 70 for 1,1-DCE, 8,500 for 1,1-DCEA, 50 for 1,2-DCEA, 50 for DCM, 50 for PCE, 50 for TCE, 10,000 for TLN, and 20 for VC.

^3^The concentration larger than first category of groundwater pollution control standards (μg/L) which were 100 for CHCL3, 70 for cis-1,2-DCE, 7 for 1,1-DCE, 850 for 1,1-DCEA, 5 for 1,2-DCEA, 5 for DCM, 5 for PCE, 5 for TCE, 1,000 for TLN, and 2 for VC.

**Table 2:** Demographic and clinical characteristics categorized by exposure group.

Characteristics	Exposure groups (villages)			
	Highly-polluted (I and II)	Moderately-polluted (III, IV, and V)	Non-polluted (VI)	P value
N	840	1,846	1,746	
		**N (%) or mean ± SD**		
Age (yrs)				<0.001
≥18–<40	298 (35.5)	721 (39.1)	773 (44.3)	
≥40–<65	423 (50.4)	916 (49.6)	844 (48.3)	
≥65	119 (14.2)	209 (11.3)	129 (7.4)	
Gender				0.031
male	404 (48.1)	790 (42.8)	793 (45.4)	
female	436 (51.9)	1,056 (57.2)	953 (54.6)	
Education level				<0.001
<high school	69 (8.5)	127 (7.1)	58 (3.4)	
high school	520 (63.7)	1054 (58.6)	929 (53.9)	
>high school	227 (27.8)	617 (34.3)	738 (42.8)	
Marital status				<0.001
unmarried or divorced	271 (33.5)	578 (32.3)	442 (25.8)	
married	539 (66.5)	1,213 (67.7)	1,268 (74.2)	
Sources of drinking water				<0.001
running water	759 (94.6)	1,587 (89.4)	1,636 (95.7)	
ground water	43 (5.4)	189 (10.6)	74 (4.3)	
Smoking				0.001
no	589 (71.3)	1,410 (78.0)	1,313 (75.7)	
yes	237 (28.7)	398 (22.0)	422 (24.3)	
Alcohol drinking				0.426
no	634 (77.1)	1,427 (79.4)	1,361 (78.9)	
yes	188 (22.9)	371 (20.6)	365 (21.1)	
Betel nut chewing				0.001
no	776 (93.8)	1,741 (95.8)	1,688 (97.1)	
yes	51 (6.2)	77 (4.2)	51 (2.9)	
Vegetarian				0.141
no	688 (85.9)	1,575 (88.6)	1,482 (87.5)	
yes	113 (14.1)	202 (11.4)	212 (12.5)	
Diabetes				0.004
no	715 (89.7)	1,586 (90.8)	1,583 (93.3)	
yes	82 (10.3)	160 (9.2)	114 (6.7)	
Hypertension				<0.001
no	460 (63.0)	707 (60.0)	1,255 (74.7)	
yes	270 (37.0)	472 (40.0)	425 (25.3)	
Anti-HCV				0.080
negative	788 (95.2)	1,753 (96.9)	1,644 (95.8)	
positive	40 (4.8)	57 (3.1)	72 (4.2)	
BMI (kg/m2)	24.45 ± 6.14	23.83 ± 3.75	24.05 ± 3.85	0.014

Abbreviation: BMI = body mass index; SD = standard deviation; HCV = hepatitis C virus.

**Table 3:** Clinical indicators of renal function by exposure group.

Variables	Exposure groups (villages)			
	Highly-polluted (I and II)	Moderately-polluted (III, IV, and V)	Non-polluted (VI)	P value
N	840	1,846	1,746	
		**N (%) or mean ± SD**		
Proteinuria				0.001
No	613 (75.5)	1,425 (80.4)	1,385 (82.0)	
Yes	199 (24.5)	347 (19.6)	303 (18.0)	
eGFR (ml/min/1.73 m^2^)^1^	88.92 ± 19.60	91.88 ± 19.70	93.19 ± 17.84	<0.001
eGFR <60 (ml/min/1.73 m^2^)				<0.001
No	779 (94.2)	1,735 (95.8)	1,678 (97.8)	
Yes	48 (5.8)	76 (4.2)	38 (2.2)	
Serum BUN (mg/dl)	14.20 ± 4.17	13.72 ± 4.16	13.45 ± 3.67	<0.001
Serum BUN (mg/dl)^2^				0.175
Normal	775 (93.7)	1,717 (94.8)	1,638 (95.5)	
Abnormal	52 (6.3)	94 (5.2)	78 (4.5)	
Serum creatinine (mg/dl)	0.90 ± 0.27	0.87 ± 0.33	0.85 ± 0.19	<0.001
Serum creatinine (mg/dl)^2^				0.017
Normal	785 (94.9)	1,738 (96.0)	1,667 (97.1)	
Abnormal	42 (5.1)	73 (4.0)	49 (2.9)	
Serum uric acid (mg/dl)	5.67 ± 1.50	5.58 ± 1.47	5.47 ± 1.41	0.005
Serum uric acid (mg/dl)^2^				0.076
Normal	786 (95.0)	1,745 (96.4)	1,662 (96.9)	
Abnormal	41 (5.0)	66 (3.6)	54 (3.1)	

Abbreviation: BUN = Blood urea nitrogen; eGFR = estimated glomerular filtration rate.

^1^eGFR is calculated based on the Modification of Diet in Renal Disease (MDRD) calculator-extended version (adjusted by age, gender, serum creatinine and race), unit as ml/min/1.73 m^2^.

^2^Abnormal values in serum were >20 mg/dl for BUN, >1.27 mg/dl in males and >1.03 mg/dl in females for creatinine, and >8.3 mg/dl for uric acid based on the clinical cut-off points used by Kaohsiung Chang-Gung Memorial Hospital.

**Table 4:** Relationship between indicators of renal function impairment and exposure group in logistic regression models.

Exposure groups	Proteinuria		OR (95% CI)	AOR (95% CI)^1^	AOR (95% CI)^2^
	Abnormal	Normal			
	(N = 849)	(N = 3,423)			
Non-polluted village	303 (18.0)	1,385 (82.0)	1.00	1.00	1.00
Moderately-polluted villages	347 (19.6)	1,425 (80.4)	1.11 (0.94–1.32)	1.03 (0.85–1.23)	0.92 (0.73–1.15)
Highly-polluted villages	199 (24.5)	613 (75.5)	1.48*** (1.21–1.82)	1.33* (1.07–1.66)	1.46** (1.15–1.85)
**Exposure groups**	**eGFR <60 (ml/min/1.73 m**^**2**^)		**OR (95% CI)**	**AOR (95% CI)**^3^	**AOR (95% CI)**^4^
	**Yes**	**No**			
	**(N = 162)**	**(N = 4,192)**			
Non-polluted village	38 (2.2)	1,678 (97.8)	1.00	1.00	1.00
Moderately-polluted villages	76 (4.2)	1,735 (95.8)	1.93** (1.30–2.87)	1.86** (1.21–2.86)	1.74* (1.02–2.97)
Highly-polluted villages	48 (5.8)	779 (94.2)	2.72*** (1.76–4.20)	1.94** (1.18–3.18)	1.89* (1.09–3.28)

Abbreviations: AOR = adjusted odds ratio; BMI = body mass index; CI = confidence interval; eGFR = estimated glomerular filtration rate; HCV = hepatitis C virus; OR = odds ratio.

*p < 0.05, **p < 0.01, ***p < 0.001.

^1^Adjusting for age, sex, education level, marriage, smoking, alcohol drinking, betel nut chewing, vegetarian, and sources of drinking water.

^2^Adjusting for age, sex, education level, marriage, smoking, alcohol drinking, betel nut chewing, vegetarian, sources of drinking water, BMI, diabetes, hypertension, anti-HCV, and serum uric acid.

^3^Adjusting for education level, marriage, smoking, alcohol drinking, betel nut chewing, vegetarian, and sources of drinking water.

^4^Adjusting for education level, marriage, smoking, alcohol drinking, betel nut chewing, vegetarian, sources of drinking water, BMI, diabetes, hypertension, anti-HCV, and serum uric acid.

## References

[b1] Kistemann T., Hundhausen J., Herbst S., Claßen T. & Färber H. Assessment of a groundwater contamination with vinyl chloride (VC) and precursor volatile organic compounds (VOC) by use of a geographical information system (GIS). Int J Hyg Environ Health. 211, 308–317 (2008). 17869578 10.1016/j.ijheh.2007.02.011

[b2] Höhener P., Werner D., Baisiger C. & Pasteris G. Worldwide occurrence and fate of chlorofluorocarbons in groundwater. Crit Rev Environ Sci Technol. 33, 1–29 (2003).

[b3] Ahmad S. A. . Arsenic in drinking water and pregnancy outcomes. Environ Health Perspect. 109, 629–631 (2001). 11445518 10.1289/ehp.01109629 PMC1240346

[b4] Zhe S. Y., Shi Z. N., Zhang D. P., Huang J. Q. & Yu G. P. Investigation of the effect of drinking water polluted by petroleum chemical industrial waste water on village residents. Huanjing Yu Jiankang Zazhi (J Environ Health). 8, 193–195 (1991).

[b5] Hu J. R. Status of ground water in the sewage storage project in the upper reach of Baiyangdian and its influences on the health of residents. Huanjing Yu Jiankang Zazhi (J Environ Health) 14, 3–5 (1994).

[b6] Griffith J., Duncan R. C., Riggan W. B. & Pellom A. C. Cancer mortality in US counties with hazardous waste sites and ground water pollution. Arch Environ Health. 44, 69–74 (1989). 2930248 10.1080/00039896.1989.9934378

[b7] Lee L. J. H. . Increased mortality odds ratio of male liver cancer in a community contaminated by chlorinated hydrocarbons in groundwater. Occup Environ Med. 60, 364–369 (2003). 12709523 10.1136/oem.60.5.364 PMC1740539

[b8] Mallin K. Investigation of a bladder cancer cluster in northwestern Illinois. Am J Epidemiol. 132 (supp1), 96–106 (1990). 2356842 10.1093/oxfordjournals.aje.a115795

[b9] Najem G. R., Strunck T. & Feuerman M. Health effects of a Superfund hazardous chemical waste disposal site. Am J Prev Med. 10, 151–155 (1994). 7917441

[b10] Logue J. N., Stroman R. M., Reid D., Hayes C. W. & Sivarajah K. Investigation of potential health effects associated with well water chemical contamination in Londonderry Township, Pennsylvania, USA. Arch Environ Health. 40, 155–160 (1985). 4026385 10.1080/00039896.1985.10545909

[b11] Schattner A., Geltner D. & Bentwich Z. Immunocomplex nephritis and myopathy in a patient who works with vinyl chloride. Arch Intern Med. 143, 843–843 (1983). 6220684

[b12] Mutti A. . Nephropathies and exposure to perchloroethylene in dry-cleaners. The Lancet. 340, 189–193 (1992). 10.1016/0140-6736(92)90463-d 1353133

[b13] Chiu C. Y. Y. H. F. . Cancer mortality and residence near petrochemical industries in Taiwan. J Toxicol Environ Health Part A. 50, 265–274 (1997). 9055875

[b14] Kelsh M. A., Alexander D. D., Mink P. J. & Mandel J. H. Occupational trichloroethylene exposure and kidney cancer: a meta-analysis. Epidemiology. 21, 95–102 (2010). 20010212 10.1097/EDE.0b013e3181c30e92

[b15] Verplanke A. J., Leummens M. H. & Herber R. F. Occupational exposure to tetrachloroethene and its effects on the kidneys. J Occup Environ Med. 41, 11–16 (1999). 9924715 10.1097/00043764-199901000-00003

[b16] Calvert G. M., Ruder A. M. & Petersen M. R. Mortality and end-stage renal disease incidence among dry cleaning workers. Occup Environ Med. 68, 709–716 (2011). 21172794 10.1136/oem.2010.060665

[b17] Ward A. M. Evidence of an immune complex disorder in vinyl chloride workers. Proc R Soc Med. 69, 289 (1976). 131319 10.1177/003591577606900421 PMC1864494

[b18] Nagaya T., Ishikawa N. & Hata H. Urinary total protein and beta-2- microglobulin in workers exposed to trichloroethylene. Environ Res. 50, 86–92 (1989). 2676510 10.1016/s0013-9351(89)80050-7

[b19] Rasmussen K., Brogren C. H. & Sabroe S. Subclinical effects on liver and kidney function and solvent exposure. Int Arch Occup Environ Health. 64, 445–448 (1993). 8458661 10.1007/BF00517951

[b20] Brüning T. . Glutathione transferase alpha as a marker for tubular damage after trichloroethylene exposure. Arch toxicol 73, 246–254 (1999). 10463390 10.1007/s002040050613

[b21] Henschler D. . Increased incidence of renal cell tumors in a cohort of cardboard workers exposed to trichloroethylene. Arch Toxicol. 69, 291–299 (1995). 7654132 10.1007/s002040050173

[b22] Vamvakas S. . Renal cell cancer correlated with occupational exposure to trichloroethylene. J Cancer Res Clin Oncol. 124, 374–82 (1998). 9719500 10.1007/s004320050186 PMC12201514

[b23] Lash L. H., Parker J. C. & Scott C. S. Modes of action of trichloroethylene for kidney tumorigenesis. Environ Health Perspect. 108, 225–240 (2000). 10.1289/ehp.00108s2225 PMC1637767 10807554

[b24] Lash L. H. & Parker J. C. Hepatic and renal toxicities associated with perchloroethylene. Pharmacol Rev. 53, 177–208 (2001). 11356983

[b25] McKone T. E. Household exposure models. Toxicol Lett. 49, 321–339 (1989). 2690408 10.1016/0378-4274(89)90040-4

[b26] Lee L. J. H. . Health risk assessment on residents exposed to chlorinated hydrocarbons contaminated in groundwater of a hazardous waste site. J Toxicol Environ Health Part A. 65, 219–235 (2002). 11911487 10.1080/15287390252800828

[b27] McKone T. E. Human exposure to volatile organic compounds in household tap water: the indoor inhalation pathway. Environ Sci Technol. 21, 1194–1201 (1987).

[b28] Brown H. S., Bishop D. R. & Rowan C. A. The role of skin absorption as a route of exposure for volatile organic compounds (VOCs) in drinking water. Am J Public Health. 74, 479–484 (1984). 6711723 10.2105/ajph.74.5.479 PMC1651599

[b29] Yu C. L. . Residential exposure to petrochemicals and the risk of leukemia: using geographic information system tools to estimate individual-level residential exposure. Am J Epidemiol. 164, 200–207 (2006). 16754633 10.1093/aje/kwj182

[b30] Lin M. Y. . Areca users in combination with tobacco and alcohol use are associated with younger age of diagnosed esophageal cancer in Taiwanese men. PLoS One 6, e25347 (2011). 22039411 10.1371/journal.pone.0025347 PMC3198438

[b31] Froissart M., Rossert J., Jacquot C., Paillard M. & Houillier P. Predictive performance of the Modification of Diet in Renal Disease and Cockcroft-Gault equations for estimating renal function. J Am Soc Nephrol. 16, 763–73 (2005). 15659562 10.1681/ASN.2004070549

