# Supplementary material for: Residential exposure to chlorinated hydrocarbons from groundwater contamination and the impairment of renal function-An ecological study
Source: Sci Rep. 2017 Jan 9;7:40283. doi: 10.1038/srep40283 (PMC5220340; doi:10.1038/srep40283)
Supplement: Supplementary Information [file srep40283-s1.pdf]

Supplementary Material

Residential exposure to chlorinated hydrocarbons from groundwater contamination and the impairment of renal function-An ecological study

Hui-Ming Chen,[1,2] Ming-Tsang Wu[1,3,4,5]

[1]Department of Public Health, College of Health Sciences, Kaohsiung Medical University, Kaohsiung, Taiwan

[2]Department of Family Medicine and Occupational Medicine, Kaohsiung Chang-Gang Memorial Hospital, Kaohsiung, Taiwan

[3]Research Center of Environmental Medicine, Kaohsiung Medical University, Kaohsiung, Taiwan

[4]Department of Family Medicine, Kaohsiung Medical University Hospital, Kaohsiung Medical University, Kaohsiung, Taiwan

[5]Center of Environmental and Occupational Medicine, Kaohsiung Municipal Hsiao-Kang Hospital, Kaohsiung Medical University, No.482, Shanming Road, Kaohsiung 812, Taiwan

eTable 1. Other demographic and clinical characteristics by exposure group.

Variables	Exposure groups (villages) Highly-polluted (I and II)	Moderately-polluted (III, IV, and V)	Non-polluted (VI)	P value
N	840	1,846	1,746	
		**N (%) or mean ± SD**		
Body height (cm)	162.5 ± 9.5	163.0 ± 8.6	163.4 ± 8.7	0.053
Body weight (kg)	64.4 ± 13.0	63.5 ± 12.1	64.5 ± 13.1	0.121
Waist (cm)	81.7 ± 11.4	79.0 ± 10.7	80.0 ± 10.8	< 0.001
BP (mmHg)				
SBP	129.6 ± 19.6	127.2 ± 19.7	124.4 ± 18.1	< 0.001
DBP	75.9 ± 11.3	74.2 ± 11.3	72.8 ± 11.3	< 0.001
Blood routine				
WBC ($10^3/\mu L$)	6.48 ± 1.71	6.41 ± 1.63	6.48 ± 1.71	0.413
RBC ($10^6/\mu L$)	4.90 ± 0.53	4.87 ± 0.56	4.92 ± 0.54	0.009
HGB (g/dL)	14.26 ± 1.59	14.18 ± 1.58	14.34 ± 1.57	0.010
HCT (%)	43.52 ± 4.12	43.01 ± 4.06	43.41 ± 4.06	0.002
MCV (fl)	89.24 ± 7.58	88.88 ± 7.49	88.57 ± 7.01	0.093
MCH (pg)	29.23 ± 2.93	29.30 ± 2.94	29.26 ± 2.78	0.811
MCHC (g/dL)	32.71 ± 1.11	32.92 ± 1.08	32.99 ± 1.05	< 0.001
PLT (10^3/uL)	252.93 ± 62.73	254.45 ± 60.89	256.76 ± 62.71	0.294
Hepatitis B antigen				0.215
Negative	728(87.9)	1571(86.8)	1467(85.5)	
Positive	100(12.1)	239(13.2)	249(14.5)	
Liver function				
GOT (IU/L)	26.60 ± 18.39	25.70 ± 18.47	25.08 ± 15.54	0.115
GPT (IU/L)	27.08 ± 27.39	26.58 ± 30.57	26.12 ± 22.61	0.696
r-GT (IU/L)	18.29 ± 21.74	17.47 ± 24.33	16.31 ± 19.87	0.077
Cardiometabolic indicators				
Glucose (mg/dL)	96.41 ± 32.02	96.15 ± 29.59	91.98 ± 20.03	< 0.001
T-cholesterol(mg/dL)	198.15 ± 39.50	196.26 ± 36.66	195.83 ± 35.58	0.352
Triglyceride (mg/dL)	125.66 ± 87.55	125.37± 121.60	123.68 ±108.07	0.872

Thyroid function				
T4 (μg/dL)	8.18 ± 1.42	7.98 ± 1.44	8.06 ± 1.36	0.004
T3 (μg/dL)	108.92 ± 21.53	110.63 ± 25.29	109.49 ± 19.26	0.121
TSH (μIU/mL)	1.85 ± 2.08	1.97 ± 2.62	1.89 ± 3.46	0.561
Tumor markers				
CEA (ng/ml)	2.43 ± 1.89	2.43 ± 1.71	2.22 ± 2.08	0.001
CA125 (u/ml)	17.63± 22.55	16.12 ± 15.74	17.99 ± 20.19	0.074
CA19-9 (u/ml)	10.75 ± 13.27	10.82 ± 12.23	10.29 ± 18.59	0.687
PSA (u/ml)	1.15 ± 1.52	1.06 ± 1.28	1.01 ± 1.21	0.263
AFP (ng/ml)	3.58 ± 9.71	4.09 ± 15.28	4.36 ± 21.17	0.554

Abbreviation: AFP: alpha-fetoprotein; BP: blood pressure; CA125: cancer antigen 125; CA19-9: cancer antigen 19-9; CEA: carcinoembryonic antigen; DBP: diastolic blood pressure; GOT: glutamate oxaloacetate transaminase; GPT: glutamic-pyruvic transaminase; HCT: hematocrit; HGB: hemoglobin; MCH: mean cell hemoglobin; MCHC: mean corpuscular hemoglobin concentration; MCV: mean cell volume; PLT: platelets; PSA: prostatic specific antigen; RBC: red blood cell count; r-GT: r-glutamyl transpeptidase; SBP: systolic blood pressure; WBC: white blood cell count; T3: triiodothyronine; T4: thyroxin; TSH: thyroid - stimulating hormone.

eTable 2. Clinical indicators of renal function by village.

	Villages						
**Variables**	**I**	**II**	**III**	**IV**	**V**	**VI**	**P value**
N	352	488	445	492	909	1,746	
			**N (%) or mean ± SD**				
Proteinuria							< 0.001
No	237 (70.5)	376 (79.0)	327 (75.3)	367 (78.8)	731 (83.8)	1,385 (82.0)	
Yes	99 (29.5)	100 (21.0)	107 (24.7)	99 (21.2)	141 (16.2)	303 (18.0)	
eGFR (ml/min/1.73 m2)[1]	89.01 ± 19.54	88.86 ± 19.67	90.15 ± 20.14	89.52 ± 18.79	94.00 ± 19.76	93.19 ± 17.84	< 0.001
eGFR < 60 (ml/min/1.73 m2)							< 0.001
No	322 (93.3)	457 (94.8)	420 (95.0)	455 (95.2)	860 (96.5)	1,678 (97.8)	
Yes	23 (6.7)	25 (5.2)	22 (45.0)	23 (4.8)	31 (3.5)	38 (2.2)	
Serum BUN (mg/dl)	14.30 ± 4.36	14.13 ± 4.04	14.16 ± 4.08	13.79 ± 4.33	13.47 ± 4.10	13.45 ± 3.67	< 0.001
Serum creatinine (mg/dl)	0.90 ± 0.25	0.90 ± 0.28	0.88 ± 0.26	0.88 ± 0.28	0.86 ± 0.38	0.85 ± 0.19	0.003
Serum uric acid (mg/dl)	5.58 ± 1.47	5.73 ± 1.53	5.52 ± 1.43	5.70 ± 1.57	5.54 ± 1.43	5.47 ± 1.41	0.005

Abbreviation: BUN = Blood urea nitrogen; eGFR = estimated glomerular filtration rate.

[1]eGFR is calculated based on the Modification of Diet in Renal Disease (MDRD) calculator-extended version (adjusted by age, gender, serum creatinine and race), unit as ml/min/1.73m^2.

eTable 3. Relationship between indicators of renal function impairment and different villages in logistic regression models.

Villages	Proteinuria Abnormal (N = 849)	Proteinuria Normal (N = 3,423)	OR (95%CI)	AOR (95%CI)[1]	AOR (95%CI)[2]
VI	303 (18.0)	1,385 (82.0)	1.00	1.00	1.00
V	141 (16.2)	731 (83.8)	0.88 (0.71-1.1.0)	0.87 (0.69-1.10)	0.84 (0.65-1.08)
III + IV	206 (22.9)	694 (77.1)	1.36** (1.11-1.66)	1.18 (0.95-1.46)	1.27 (0.86-1.87)
II	100 (21.0)	376 (79.0)	1.22 (0.94-1.57)	1.09 (0.83-1.44)	1.29 (0.96-1.73)
I	99 (29.5)	237 (70.5)	1.91*** (1.46-2.49)	1.74*** (1.30-2.34)	1.70** (1.25-2.32)

Villages	eGFR < 60 (ml/min/1.73 m^2) Yes (N = 162)	eGFR < 60 (ml/min/1.73 m^2) No (N = 4,192)	OR (95%CI)	AOR (95%CI)[3]	AOR (95%CI)[4]
VI	38 (2.2)	1,678 (97.8)	1.00	1.00	1.00
V	31 (3.5)	860 (96.5)	1.59 (0.98-2.58)	1.79* (1.07-3.01)	1.58 (0.89-2.82)
III + IV	45 (4.9)	875 (95.1)	2.27*** (1.46-3.52)	1.91** (1.18-3.09)	2.38* (1.04-5.43)
II	25 (5.2)	457 (94.8)	2.42** (1.44-4.04)	1.81* (1.01-3.24)	1.95* (1.01-3.74)
I	23 (6.7)	322 (93.3)	3.15*** (1.85-5.37)	2.12* (1.13-3.98)	1.82 (0.90-3.67)

Abbreviations: AOR = adjusted odds ratio; BMI = body mass index; CI, confidence interval; eGFR = estimated glomerular filtration rate; HCV = hepatitis C virus; OR = odds ratio.

[1]Adjusting for age, sex, education level, marriage, smoking, alcohol drinking, betel nut chewing, vegetarian, and sources of drinking water.

[2]Adjusting for age, sex, education level, marriage, smoking, alcohol drinking, betel nut chewing, vegetarian, sources of drinking water, BMI, diabetes, hypertension, anti-HCV, and serum uric acid,

[3]Adjusting for education level, marriage, smoking, alcohol drinking, betel nut chewing, vegetarian, and sources of drinking water.

[4]Adjusting for education level, marriage, smoking, alcohol drinking, betel nut chewing, vegetarian, sources of drinking water, BMI, diabetes, hypertension, anti-HCV, and serum uric acid.

*$p < 0.05$, **$p < 0.01$, ***$p < 0.001$.

eTable 4. Relationship between indicators of renal function impairment and exposure group dichotomized by age of 65 years old in logistic regression models.

Age 18-64 yrs	Proteinuria				
Exposure groups	Abnormal (N = 699)	Normal (N = 3,133)	OR (95%CI)	AOR (95%CI)[1]	AOR (95%CI)[2]
Non-polluted village	260 (16.7)	1,301 (83.3)	1.00	1.00	1.00
Moderately-polluted villages	286 (18.2)	1,289 (81.8)	1.11 (0.92-1.34)	1.06 (0.87-1.29)	0.92 (0.73-1.18)
Highly-polluted villages	153 (22.0)	543 (78.0)	1.41**(1.13-1.76)	1.32*(1.03-1.67)	1.45**(1.13-1.87)

	eGFR < 60 (ml/min/1.73 m^2)				
Exposure groups	Yes (N = 68)	No (N = 3837)	OR (95%CI)	AOR (95%CI)[3]	AOR (95%CI)[4,5]
Non-polluted village	20 (1.3)	1,569 (98.7)	1.00	1.00	1.00
Moderately-polluted villages	34 (2.1)	1,573 (97.9)	1.70 (0.97-2.96)	1.91* (1.03-3.55)	1.35 (0.64-2.86)
Highly-polluted villages	14 (2.0)	695 (98.0)	1.58(0.79-3.15)	1.71 (0.80-3.68)	1.57 (0.69-3.57)

Age ≥ 65 yrs	Proteinuria				
Exposure groups	Abnormal (N = 150)	Normal (N = 290)	OR (95%CI)	AOR (95%CI)[1]	AOR (95%CI)[2]
Non-polluted village	43 (33.9)	84 (66.1)	1.00	1.00	1.00
Moderately-polluted villages	61 (31.0)	136 (69.0)	0.88 (0.55-1.41)	0.81 (0.46-1.40)	0.77 (0.39-1.55)
Highly-polluted villages	46 (39.7)	70 (60.3)	1.28 (0.76-2.17)	1.34 (0.73-2.45)	1.58 (0.81-3.07)

	eGFR < 60 (ml/min/1.73 m^2)				
Exposure groups	Yes (N = 94)	No (N = 355)	OR (95%CI)	AOR (95%CI)[3]	AOR (95%CI)[4,6]
Non-polluted village	18 (14.2)	109 (85.8)	1.00	1.00	1.00
Moderately-polluted villages	42 (20.6)	162 (79.4)	1.57 (0.86-2.87)	1.54 (0.80-2.94)	1.89 (0.83-4.31)
Highly-polluted villages	34 (28.8)	84 (71.2)	2.45** (1.30-4.64)	1.65 (0.81-3.36)	2.00 (0.88-4.54)

Abbreviations: AOR = adjusted odds ratio; BMI = body mass index; CI = confidence interval; eGFR = estimated glomerular filtration rate; HCV = hepatitis C virus; OR = odds ratio.

[1]Adjusting for age, sex, education level, marriage, smoking, alcohol drinking, betel nut chewing, vegetarian, and sources of drinking water.

[2]Adjusting for age, sex, education level, marriage, smoking, alcohol drinking, betel nut chewing, vegetarian, sources of drinking water, BMI, diabetes, hypertension, anti-HCV, and serum uric acid.

[3]Adjusting for education level, marriage, smoking, alcohol drinking, betel nut chewing,

vegetarian, and sources of drinking water.

[4]Adjusting for education level, marriage, smoking, alcohol drinking, betel nut chewing, vegetarian, sources of drinking water, BMI, diabetes, hypertension, anti-HCV, and serum uric acid.

[5]Trend test, $p = 0.262$.

[6]Trend test, $p = 0.102$.

$^{*}p < 0.05$, $^{**}p < 0.01$, $^{***}p < 0.001$

eTable 5. Relationship between indicators of renal function impairment and exposure group dichotomized by the history of diabetes in logistic regression models.

**Without diabetes**	**Proteinuria**				
**Exposure groups**	**Abnormal (N = 766)**	**Normal (N = 3,268)**	**OR (95%CI)**	**AOR (95%CI)[1]**	**AOR (95%CI)[2]**
Non-polluted village	278 (17.2)	1,337 (82.8)	1.00	1.00	1.00
Moderately-polluted villages	317 (19.0)	1,350 (81.0)	1.13 (0.95-1.35)	1.05 (0.87-1.28)	0.93 (0.74-1.18)
Highly-polluted villages	171 (22.7)	581 (77.3)	1.42**(1.14-1.75)	1.29*(1.07-1.63)	1.42**(1.11-1.81)

	**eGFR < 60 (ml/min/1.73 m^2)**				
**Exposure groups**	**Yes (N = 126)**	**No (N = 3,984)**	**OR (95%CI)**	**AOR (95%CI)[3]**	**AOR (95%CI)[4,5]**
Non-polluted village	31 (1.9)	1,611 (98.1)	1.00	1.00	1.00
Moderately-polluted villages	60 (3.5)	1,640 (96.5)	1.90** (1.23-2.95)	2.28** (1.43-3.64)	1.73 (1.00-2.98))
Highly-polluted villages	35 (4.6)	733 (95.4)	2.48***(1.52-4.06)	2.37**(1.37-4.10)	1.99* (1.12-3.55)

**With diabetes**	**Proteinuria**				
**Exposure groups**	**Abnormal (N = 78)**	**Normal (N = 132)**	**OR (95%CI)**	**AOR (95%CI)[1]**	**AOR (95%CI)[2]**
Non-polluted village	25 (35.7)	45 (64.3)	1.00	1.00	1.00
Moderately-polluted villages	28 (31.5)	61 (68.5)	0.83 (0.43-1.60)	0.76 (0.35-1.67)	0.46 (0.15-1.42)
Highly-polluted villages	25 (49.0)	26 (51.0)	1.73 (0.83-3.61)	1.78 (0.77-4.11)	2.25 (0.87-5.87)

	**eGFR < 60 (ml/min/1.73 m^2)**				
**Exposure groups**	**Yes (N = 34)**	**No (N = 182)**	**OR (95%CI)**	**AOR (95%CI)[3]**	**AOR (95%CI)[4,6]**
Non-polluted village	7 (9.9)	64 (90.1)	1.00	1.00	1.00
Moderately-polluted villages	16 (16.8)	79 (83.2)	1.85 (0.72-4.78)	1.95 (0.67-5.71)	1.25 (0.21-7.52)
Highly-polluted villages	11 (22.0)	39 (78.0)	2.58 (0.92-7.21)	2.19 (0.70-6.88)	3.34 (0.78-14.39)

Abbreviations: AOR = adjusted odds ratio; BMI = body mass index; CI = confidence interval; eGFR = estimated glomerular filtration rate; HCV = hepatitis C virus; OR = odds ratio.

[1]Adjusting for age, sex, education level, marriage, smoking, alcohol drinking, betel nut chewing, vegetarian, and sources of drinking water.

[2]Adjusting for age, sex, education level, marriage, smoking, alcohol drinking, betel nut chewing, vegetarian, sources of drinking water, BMI, hypertension, anti-HCV, and serum uric acid.

[3]Adjusting for marriage, smoking, alcohol drinking, betel nut chewing, vegetarian,

and sources of drinking water.

[4]Adjusting for marriage, smoking, alcohol drinking, betel nut chewing, vegetarian, sources of drinking water, BMI, hypertension, anti-HCV, and serum uric acid.

[5]Trend test, $p = 0.015$.

[6]Trend test, $p = 0.096$.

*$p < 0.05$, **$p < 0.01$, ***$p < 0.001$

eTable 6. Relationship between serum renal function indicators and exposure group in logistic regression models.

Exposure groups	Serum BUN		OR (95%CI)	AOR (95%CI)[1]	AOR (95%CI)[2]
	Abnormal (N = 224)	Normal (N = 4,130)			
Non-polluted village	78 (4.5)	1,638 (95.5)	1.00	1.00	1.00
Moderately-polluted villages	94 (5.2)	1,717 (94.8)	1.15 (0.85-1.56)	0.96 (067-1.37)	1.00 (0.66-1.53)
Highly-polluted villages	52 (6.3)	775 (93.7)	1.41 (0.98-2.02)	0.92 (0.59-1.42)	0.93 (0.59-1.47)

Exposure groups	Serum creatinine		OR (95%CI)	AOR (95%CI)[1]	AOR (95%CI)[2,3]
	Abnormal (N = 164)	Normal (N = 4,190)			
Non-polluted village	49 (2.9)	1,667 (97.1)	1.00	1.00	1.00
Moderately-polluted villages	73 (4.0)	1,73 8 (96.0)	1.43 (0.99-2.07)	1.25 (0.82-1.91)	0.99 (0.59-1.65)
Highly-polluted villages	42 (5.1)	785 (94.9)	1.82** (1.20-2.77)	1.15 (0.70-1.90)	1.22 (0.72-2.05)

Exposure groups	Serum uric acid		OR (95%CI)	AOR (95%CI)[1]	AOR (95%CI)[2]
	Abnormal (N = 161)	Normal (N = 4,193)			
Non-polluted village	54 (3.1)	1,662 (96.9)	1.00	1.00	1.00
Moderately-polluted villages	66 (3.6)	1,745 (96.4)	1.16 (0.81-1.68)	1.05(0.71-1.57)	0.77 (0.45-1.32)
Highly-polluted villages	41 (5.0)	786 (95.0)	1.61* (1.06-2.43)	1.30 (0.82-2.06)	1.33 (0.81-2.17)

Abbreviations: AOR = adjusted odds ratio; BMI = body mass index; CI = confidence interval; HCV = hepatitis C virus; OR = odds ratio.

[1]Adjusting for age, sex, education level, marriage, smoking, alcohol drinking, betel nut chewing, vegetarian, and sources of drinking water.

[2]Adjusting for age, sex, education level, marriage, smoking, alcohol drinking, betel nut chewing, vegetarian, sources of drinking water, BMI, diabetes, hypertension, and anti-HCV.

[3]Trend test, $p = 0.498$.

*$p < 0.05$, **$p < 0.01$.
